# Clinical significance and potential pathogenesis of VCAN in adult non-cystic fibrosis bronchiectasis: a retrospective study

**DOI:** 10.1186/s12890-024-03027-4

**Published:** 2024-04-29

**Authors:** Wan-Ying Huang, Kang-Kang Hong, Rong-Quan He, Jing Luo, Zhi-Guang Huang, Chu-Yue Zhang, Yang Xu, Chong-Xi Bao, Liang-Ming Zhang, Gang Chen, Jin-Liang Kong

**Affiliations:** 1 Department of Pathology, The First Affiliated Hospital of Guangxi Medical University, Nanning, Guangxi China; 2 Ward of Pulmonary and Critical Care Medicine, Department of Respiratory Medicine, The First Affiliated Hospital of Guangxi Medical University, Nanning, Guangxi China; 3 Department of Medical Oncology, The First Affiliated Hospital of Guangxi Medical University, Nanning, Guangxi China

**Keywords:** Bronchiectasis, Pathogenesis, Transcriptomics, VCAN

## Abstract

**Background:**

The pathogenesis of adult non-cystic fibrosis (CF) bronchiectasis is complex, and the relevant molecular mechanism remains ambiguous. Versican (VCAN) is a key factor in inflammation through interactions with adhesion molecules. This study constructs a stable panoramic map of mRNA, reveals the possible pathogenesis of bronchiectasis, and provides new ideas and methods for bronchiectasis.

**Methods:**

Peripheral blood and tissue gene expression data from patients with bronchiectasis and normal control were selected by bioinformatics analysis. The expression of VCAN in peripheral blood and bronchial tissues of bronchiectasis were obtained by transcriptome sequencing. The protein expression levels of VCAN in serums were verified by the enzyme-linked immunosorbent assay (ELISA). The mRNA expression levels of VCAN in co-culture of *Pseudomonas aeruginosa* and bronchial epithelial cells were verified by real-time quantitative polymerase chain reaction (RT-qPCR). In addition, the biological function of VCAN was detected by the transwell assay.

**Results:**

The expression of VCAN was upregulated in the bronchiectasis group by sequencing analysis (*P* < 0.001). The expression of VCAN in the bronchial epithelial cell line BEAS-2B was increased in *P. aeruginosa* (*P.a*), which was co-cultured with BEAS-2B cells (*P* < 0.05). The concentration of VCAN protein in the serum of patients with bronchiectasis was higher than that in the normal control group (*P* < 0.05). Transwell experiments showed that exogenous VCAN protein induced the migration of neutrophils (*P* < 0.0001).

**Conclusions:**

Our findings indicate that VCAN may be involved in the development of bronchiectasis by increasing the migration of neutrophils and play an important role in bronchial pathogenesis.

**Supplementary Information:**

The online version contains supplementary material available at 10.1186/s12890-024-03027-4.

## Introduction

Bronchiectasis is a chronic clinical syndrome that is characterised by cough and sputum production in the abnormal thickening and dilation of the bronchial wall. The clinical course of patients with bronchiectasis is characterised by intermittent exacerbations [1]. Cystic fibrosis (CF) and non–CF are two causes of bronchiectasis. In China, approximately 1.5% of women and 1.1% of men have physician-diagnosed bronchiectasis [2]. In recent years, the prevalence of bronchiectasis is increasing worldwide [3], and the increasing socioeconomic burden has also become a global health problem [4]. Bronchiectasis is associate with inflammation and eosinophils [5], which refers to a lack of effective therapeutic strategy. Common therapeutic strategies for bronchiectasis include managing symptoms, reducing exacerbation, and slowing its progression [6]. Moreover, antibiotic misuse, opportunistic infection, and antibiotic resistance should increase economic burden and decrease the quality of life of patients [6, 7]. Accordingly, the attention given to bronchiectasis therapeutic strategy is increasing [8]. Recently, outpatient parenteral antibiotic therapy (OPAT) has become a potential treatment for bronchiectasis exacerbations management [9]. However, the pathogenesis of bronchiectasis remains ambiguous.

Versican (VCAN) belongs to the lectican protein family, which is a large chondroitin sulphate proteoglycan. Protein is a major component of the extracellular matrix [10]. The role of VCAN in cell adhesion, migration, and proliferation has been extensively investigated. VCAN is often considered an anti-adhesion molecule, whose role is increased in the changing tissue extracellular matrix of inflammatory lung disorders [11]. Thomas N. Wight et al. found that VCAN causes inflammation by interacting with a variety of growth factors and cytokines involved in regulating inflammation, thereby promoting inflammation cytokines such as TNFα, IL-8, and NFκB [12]. Therefore, the messenger RNA (mRNA) and protein levels of VCAN is closely associated with the progression and prognosis of inflammation.

**Fig. 1:**
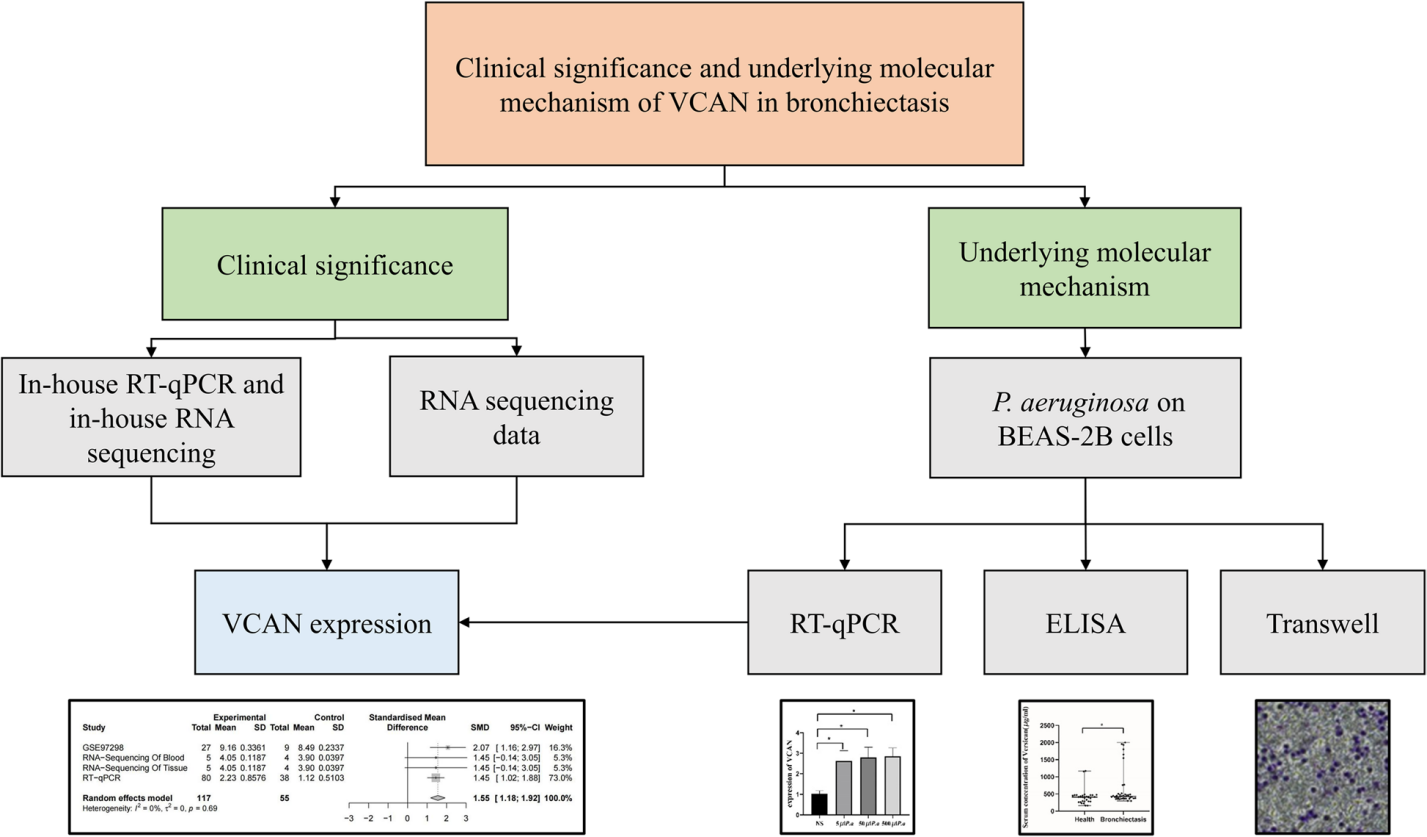
Flow chart of this paper

Overexpression of MUC5AC and MUC5B, which was observed in patients with bronchiectasis [13], was related to an increase in mucin concentrations, osmotic pressure, and elastic and viscous moduli [14]. Neutrophil extracellular trap (NET) formation is a method of host defence in multiple inflammatory diseases, which play a key marker of disease severity and treatment response in bronchiectasis [15]. However, the function between versican and neutrophil remains ambiguous. VCAN contributes to disease pathophysiology and may be a target for bronchiectasis treatment. Therefore, we explore the expression of VCAN in bronchiectasis and reveal its molecular mechanism and biological characteristics (Fig. 1).

## Methods

### Cell lines and culture conditions

The human bronchial epithelial cell line BEAS-2B was purchased from Procell (Wuhan, China), maintained in Dulbecco’s Modified Eagle Medium (DMEM) (Invitrogen, Carlsbad, CA, USA) containing 10% FBS (Invitrogen) and 100 U/ml penicillin–streptomycin. Cells were incubated at 37 °C/5% CO_2_ in a humidified chamber.

### Patients and clinical specimens collection

Peripheral blood and tissue samples were collected in this study. Peripheral blood samples for RNA sequencing were conducted by enrolling a total of five patients with bronchiectasis and four controls at the First Affiliated Hospital of Guangxi Medical University from July 2021 to Oct 2021. Tissue samples for RNA sequencing were collected from five patients (surgery was required) with bronchiectasis and relatively normal bronchus tissue samples from four patients undergoing lung space occupying surgical resection at the First Affiliated Hospital of Guangxi Medical University from Oct 2021 to July 2022. Peripheral blood samples for real-time quantitative polymerase chain reaction (RT-qPCR) analysis included 80 patients with bronchiectasis and 38 controls at the First Affiliated Hospital of Guangxi Medical University from July 2021 to Dec 2022. And the demographic and clinical characteristics of 80 patients with bronchiectasis are shown in Supplementary Table 1. Serums for enzyme-linked immunosorbent assay (ELISA) from 42 patients with bronchiectasis and 34 controls were obtained. Written informed consent was obtained from all the subjects, and the protocol was approved by the Ethical Review Committee of the First Affiliated Hospital of Guangxi Medical University. Information that can identify individual participants during or after the data collection was available and can be accessed. We confirm that all procedures were followed in accordance with relevant guidelines and regulations.

Total RNA was extracted from serum samples or tissue samples using RNAiso Blood (Takara, Japan) or RNAiso Plus (Takara, Japan) according to the manufacturer’s instructions. All RNA was stored in refrigerator at -80 °C before use.

### *P. aeruginosa* strain

*Pseudomonas aeruginosa* strain was isolated from bronchoalveolar lavage fluid or sputum of bronchiectasis patients at the First Affiliated Hospital of Guangxi Medical University. All *P. aeruginosa* (*P*.*a*) strains confirmed by matrix-assisted laser desorption/ionisation time-of-flight (MALDI-TOF) mass spectrometry. The isolation procedure was approved by the Ethical Review Committee of the First Affiliated Hospital of Guangxi Medical University. The bacteria strain was incubated in LB Broth Medium at 37 °C. After centrifugation, the *P*.*a* in the logarithmic growth phase was suspended by antibiotic-free and serum-free DMEM culture, and the absorbance was adjusted to 0.1 by an ultraviolet spectrophotometer. The concentration of bacteria was adjusted to 0.1 McI with a turbidimeter (1 × 10^8^ cfu).

### RNA extraction, cDNA synthesis and RT-qPCR

The RNA extraction, cDNA synthesis and RT-qPCR were performed as follows: 1 μg of total RNA from the peripheral blood or cells were reverse-transcribed by using the PrimeScriptTMRT Reagent Kit with gDNA Eraser (Takara, Dalian, China). The RT-qPCR assay was performed by using TB Green Premix Ex Taq TMII (Takara, Dalian, China) and the LightCycler 480III Real-time PCR system (Roche). The expression of VCAN mRNAs were normalised to the level of beta-actin mRNA. The primer sequences are presented as follows: VCAN-F 5’- CCATCTCACAAGCATCCTGTCTCAC-3’, VCAN-R 5’- CTGCCATCAGTCCAACGGAAGTC-3’, beta-actin-F 5’- CCTGGCACCCAGCACAAT -3’, and beta-actin-R 5’- GGGCCGGACTCGTCATAC -3’. The cycling conditions (40 cycles) were conducted using the following program: 95 °C for 5 s and 60 °C for 30 s. PCR reactions were performed in triplicate in a 20 μL mixture containing 10 μL of TB Green Premix Ex Tag II (2 ×), 0.8 μL of each primer (10 μM), 6.4 μL of nuclease-free water and 2 μL of template DNA. Gene expression levels were normalised to that of β-actin according to the cycle threshold (2^ − ΔΔCT^) method.

### Enzyme-linked immunosorbent assay (ELISA)

The protein level of versican from the serums were determined by an ELISA kit purchased from MLBio (Shanghai, China) according to the manufacturer’s instructions.

### Construction of mRNA-seq library and bioinformatics analysis

Messenger RNA enrichment, reverse transcription, fragmentation, adapter ligation, and PCR amplification were utilised for mRNA-seq library construction. After quality inspection of the library, the RNA Library Prep Kit (Illumina, San Diego, USA) was constructed high-throughput sequencing. The purified library was paired-end sequenced on an Illumina NovaSeq 6000 (Illumina, San Diego, USA) according to the manufacturer’s instructions.

To acquire mRNA expression in bronchiectasis, human reference genome hg38 was selected for gene sequence alignment. The mRNA expression was analysed using R software (ver. 3.5.1, the R Project for Statistical Computing). StataSE and SPSS 25.0 were employed to calculate the standardised mean difference (SMD) and receiver operating characteristic (ROC) curve. The ROC curves were used to evaluate the expression pattern of VCAN in bronchiectasis and non-bronchiectasis specimens, which were plotted for the RNA-seq dataset.

### Neutrophil isolation and transwell assay

Neutrophil samples were isolated from blood by a human neutrophil isolation kit (TBD, China). Neutrophil cells (1.0 × 10^6^ per well) were seeded in the upper chamber of 24-well invasion chambers (LABSELECT, China) coated with Matrigel. The lower chamber was filled with RPMI1640 medium (Invitrogen, Carlsbad, CA, USA) with 10% foetal bovine serum (FBS). Non-invading cells were cleaned by using a cotton-tipped swab for four hours. Migratory and invasive cells on the lower membrane surface were fixed with 1% paraformaldehyde and stained with 0.1% crystal violet. Nine random sights were photographed.

### Statistical analysis

SPSS 25.0 (SPSS Inc., Chicago, IL, USA) was used for statistical analysis. In the descriptive analyses, the mean ± standard deviation (SD) was used for normally distributed continuous variables and the median ± interquartile range (IQR) was used for continuous variables with skewed distributions. Data such as homogeneity of variance with and without a normal distribution were compared by the student’s t-test, while non-normally distributed continuous data were compared using the Mann–Whitney U test. Statistical significance was considered at *p* < 0.05.

## Results

### VCAN is upregulated in bronchiectasis peripheral blood and tissue

**Fig. 2:**
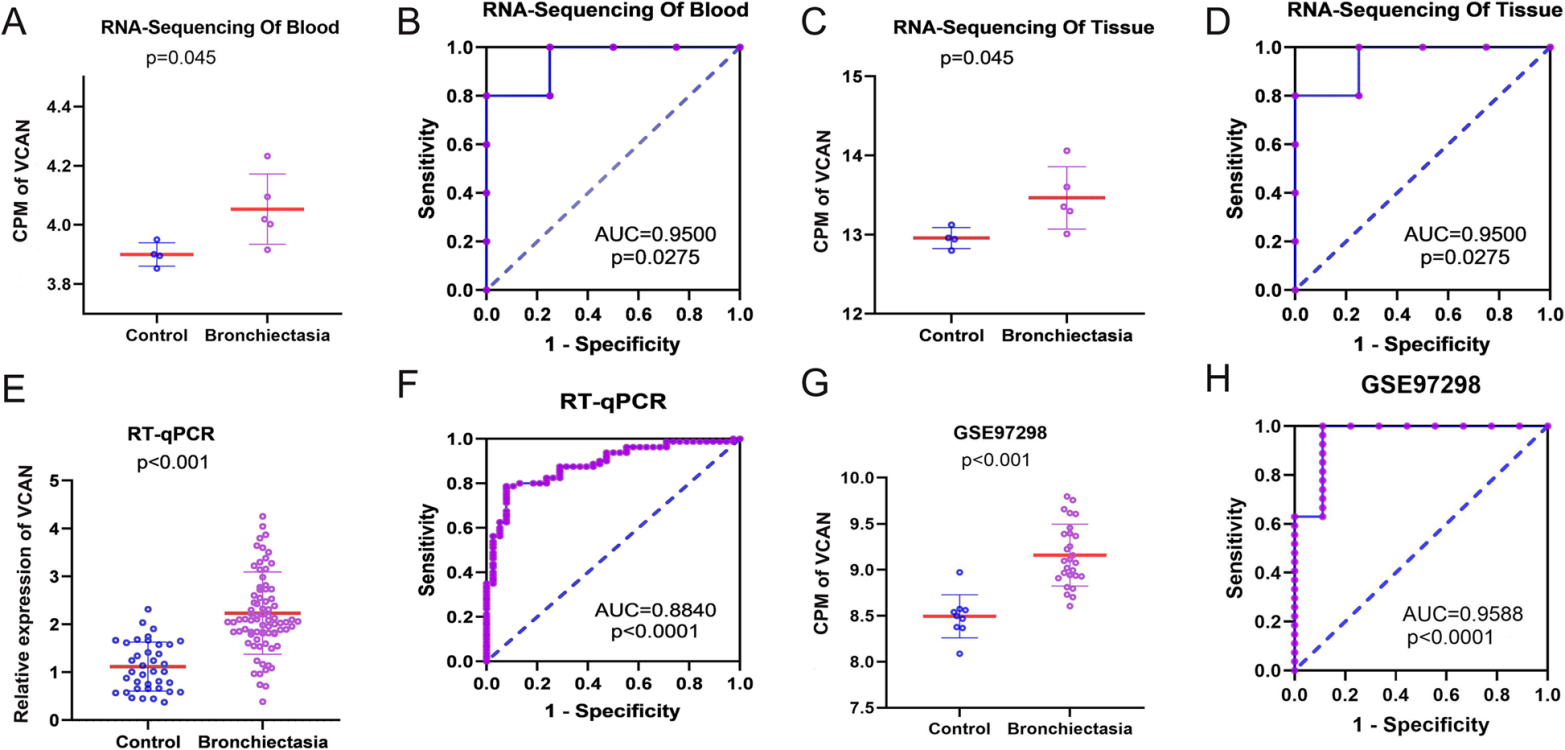
Overexpression of VCAN in bronchiectasis. **A** and **B** blood sequencing; **C** and **D** bronchiectasis tissue sequencing; **E** and **F** RT-qPCR; G and H: GSE97298 dataset. Note: CPM, Counts per million. RT-qPCR, real-time quantitative polymerase chain reaction.

To reveal a potential role for VCAN in bronchiectasis, we examined the mRNA expression of VCAN in bronchiectasis peripheral blood and bronchiectasis lung tissues from patients at the First Affiliated Hospital of Guangxi Medical University by RNA sequencing. The expression of VCAN was increased in the bronchiectasis tissues and peripheral blood (*n* = 5) compared with the controls (*n* = 4) (Fig. 2A-D). As well, according to the RT-qPCR, VCAN expression was higher in peripheral blood than the controls (Fig. 2E-F).

**Fig. 3:**
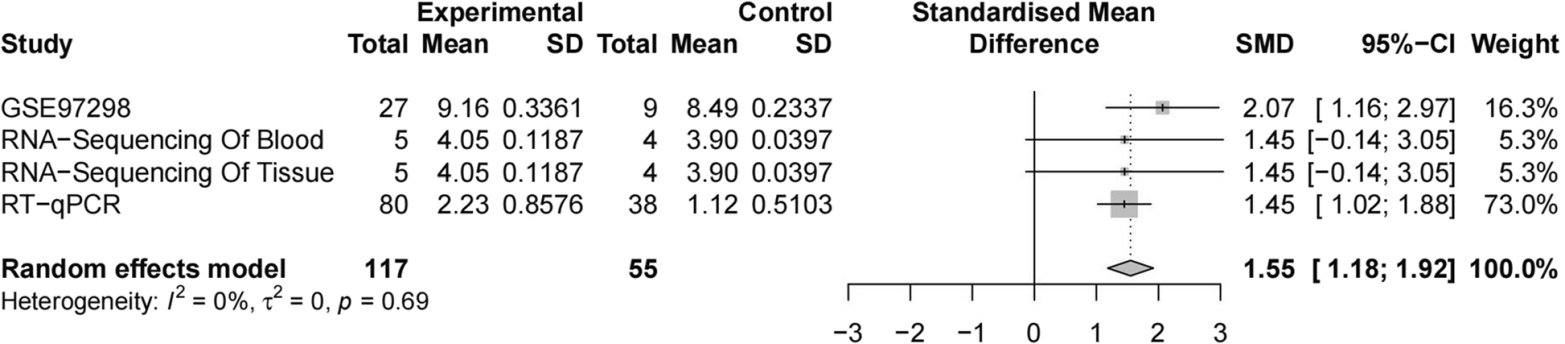
Integrated analysis of VCAN overexpression in bronchiectasis

To confirm our findings, we analysed RNA-seq data from the Gene Expression Omnibus (GEO), which is available via the following accession identifier on the dataset: GSE97298. We found VCAN was overexpressed in bronchiectasis (Fig. 2G-H); the area under the ROC curve for scatter plots of GSE97298, blood, and tissue; and RT-qPCR showed that VCAN was upregulated in bronchiectasis. As expected, VCAN was overexpressed in bronchiectasis after integrated analysis (Fig. 3).

### The *P.a* infection model is prepared

**Fig. 4:**
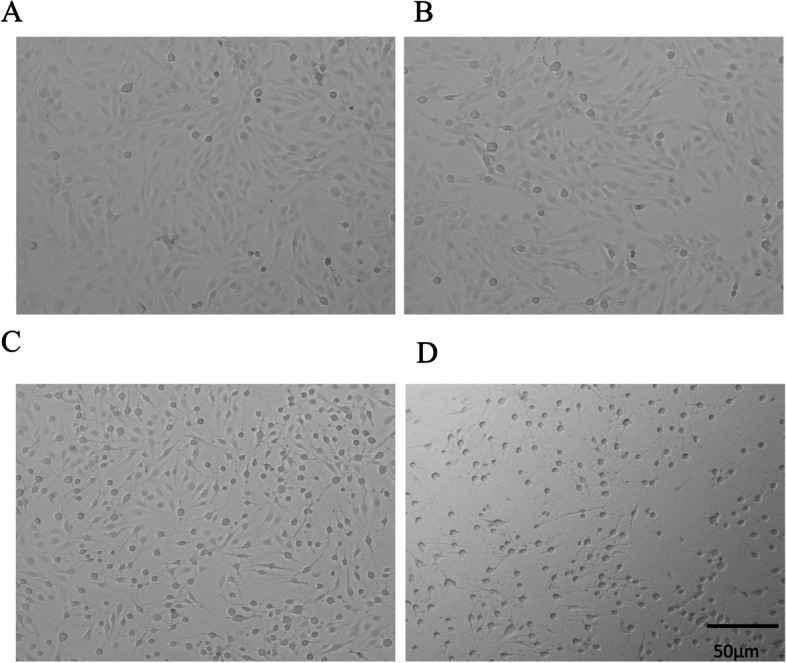
Effects of different concentrations of *P.a* on BEAS-2B cells. **A** control group; **B** 5 μl *P.a* group, treated for two hours when MOI = 1; **C** 50 μl *P.a* group, treated for two hours when MOI = 10; and **D** 500 μl *P.a* group, treated for two hours when MOI = 100

**Fig. 5:**
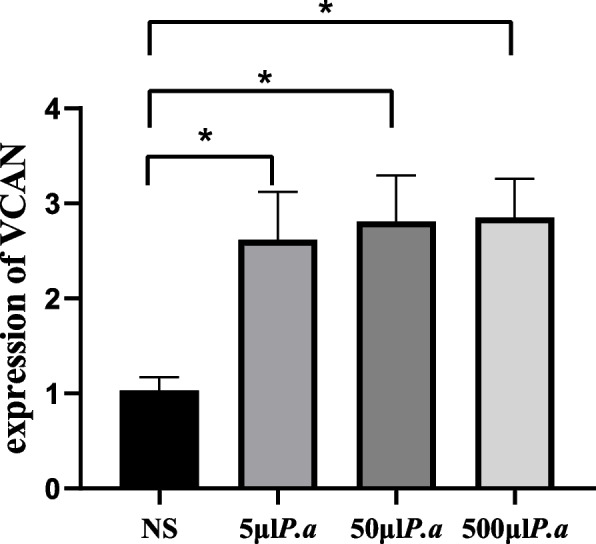
Expression of VCAN in BEAS-2B cells after intervention with different concentrations of *P.a.* Note: *P < 0.05; *P.a*, *Pseudomonas aeruginosa*

To generate the *P.a* infection model, we determine the suitable concentration of *P.a.* Different concentration groups of *P.a* were cultured with the BEAS-2B cell line (5 × 10^5^ cells), where 5 μl *P.a*, 50 μl *P.a*, and 500 μl *P.a* corresponds to a multiplicity of infection (MOI) of one, 10, and 100. The captured images showed the effects of different concentrations of *P.a* on BEAS-2B cells (Fig. 4A-D). We observed morphological injury changes in the MOI = 10 group (Fig. 4C), while the changes were the worst in the MOI = 100 group (Fig. 4D). When MOI = 10, the cells began to shrink, their morphology and refraction changed, and some cells were damaged and died. To better observe the morphological changes of cells after bacterial intervention, the concentration of MOI = 10 was selected for our study. Moreover, we confirmed that the mRNA level of VCAN was upregulated in the co-cultured group by RT-qPCR (Fig. 5).

### *P.a* infection increases the VCAN level in BEAS-2B cell

**Fig. 6:**
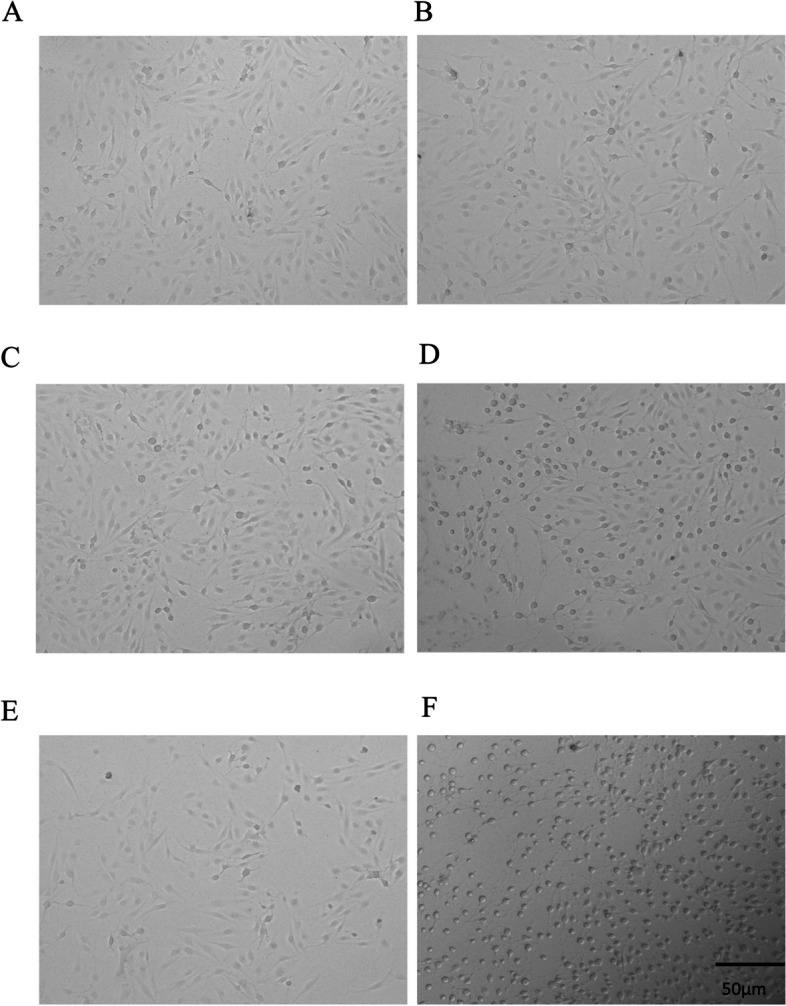
Effects of the same concentration of *P.a* on BEAS-2B cells at different intervals. **A** control group, treated for one hour; **B** 50 μl *P.a*, treated for one hour; **C** control group, treated for two hours; **D** 50 μl *P.a*, treated for two hours; **E** control group, treated for six hours; and **F** 50 μl *P.a*, treated for six hours. Note: *P.a*:* Pseudomonas aeruginosa*

**Fig. 7:**
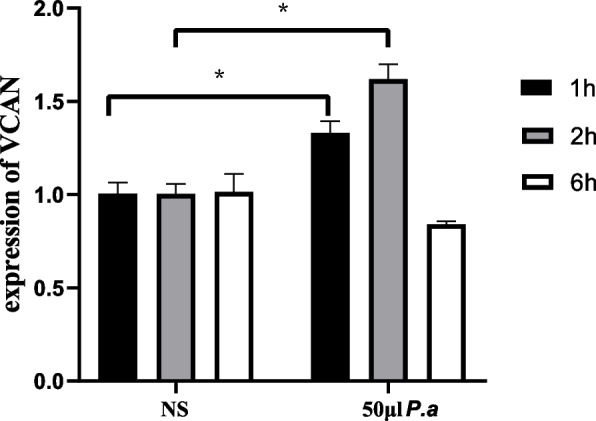
Expression of VCAN after the same concentrations of *P.a* interferes with BEAS-2B cells at different intervals. Note: *P < 0.05; *P.a*, *Pseudomonas aeruginosa*

To determine whether the effect of BEAS-2B cells co-cultured with *P.a* can increase the expression of VCAN in vitro, we detected the mRNA level of VCAN in BEAS-2B cells treated with MOI = 10 *P.a* for different intervals (one hour, two hours, and six hours, as shown in Figs. 6 and 7). Compared with the control groups (no infection with *P.a*), the expression of VCAN was significantly increased in the one-hour and two-hour groups (Fig. 7). The morphological microscope image showed that the morphological injury was mild in the one-hour group. As the interval increased, the injury worsened (Fig. 6E-F).

### The protein level of versican is upregulated in the serum of bronchiectasis patients

**Fig. 8:**
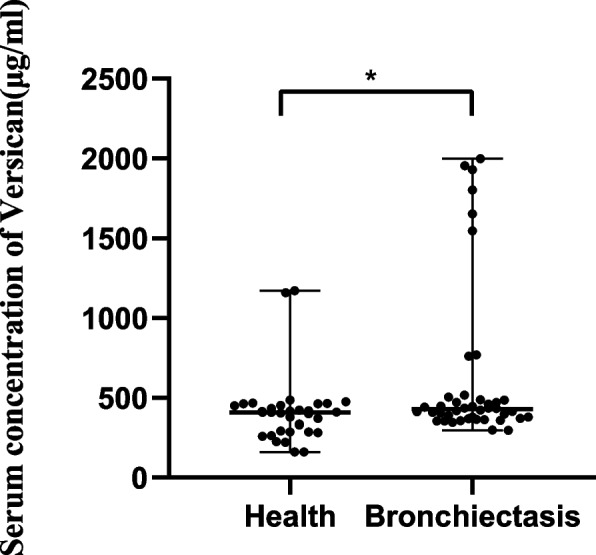
Comparison of serum VCAN concentrations in the healthy group and bronchiectasis group. Note: *P < 0.05

A prospective study was conducted by enrolling 42 patients with bronchiectasis and 34 controls. Blood samples were collected from the volunteers in the morning. Serum samples was isolated from the blood and stored at -80 °C for an ELISA. The results indicated that the concentration of versican was upregulated in bronchiectasis (Fig. 8).

### Veisican promotes the invasion of bronchiectasis neutrophils

**Fig. 9:**
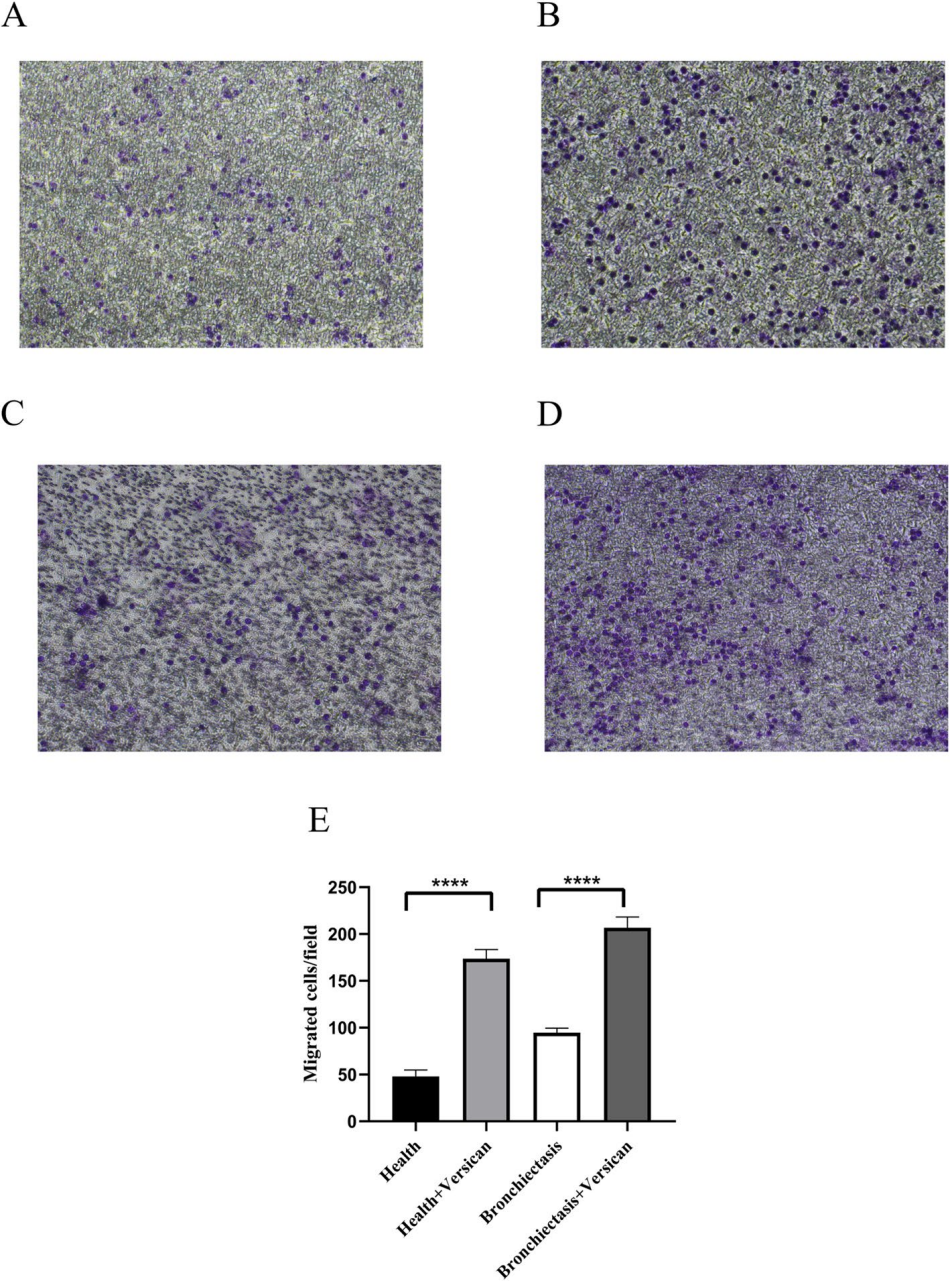
Versican enhances the migratory motility of neutrophils. **A** healthy neutrophils group; **B** healthy neutrophils + versican group; **C** bronchiectasis neutrophils group; **D** bronchiectasis neutrophils + versican group; and **E** migrated neutrophil cells per field in the four groups. Note: ****P < 0.0001

To investigate the effect of exogenous versican on the invasive capacities of bronchiectasis neutrophils, we performed a transwell assay. Neutrophil samples were isolated from the normal controls and bronchiectasis patients. The recombinant human versican protein (Cusabio, Wuhan, China) was employed in this protocol. The concentration of exogenous versican was 100 μg/ml. The protocol included four groups: the healthy neutrophils group, healthy neutrophils + versican group, bronchiectasis neutrophils group, and bronchiectasis neutrophils + versican group. We observed that more neutrophils invaded the extracellular matrix gel in contrast with the control neutrophils (Fig. 9A-E), suggesting that the migration of bronchiectasis neutrophils is attributed to versican.

## Discussion

Presently, the role of VCAN in bronchiectasis has not been reported. This study investigated the overexpression of VCAN in bronchiectasis based on the sequencing of blood and tissue and the validation of clinical samples. In addition, versican encoded by VCAN in the blood of patients with bronchiectasis was detected, and it was determined that the concentration of versican increased. The results showed that an increase in VCAN expression can lead to encoding and the translation of more versican proteins. Through in vitro cell migration experiments, it was determined that versican can induce the migration of neutrophils, indicating that VCAN plays an important role in the occurrence and development of bronchiectasis.

The VCAN gene is located on chromosome 5q14.3, and versican protein encoded by the VCAN gene is a large chondroitin sulphate proteoglycan, with at least five different subtypes (V0, V1, V2, V3, and V4). Versican is the main component of extracellular matrix, and different subtypes exhibit different biological functions. For example, Versican V1 has the ability to promote proliferation and inhibit apoptosis, while Versican V2 exhibits anti-proliferative activity. Versican V3 regulates the assembly of extracellular mechanisms and inhibits cell proliferation and migration [12]. Versican participates in cell adhesion, proliferation, migration, and angiogenesis by interacting with various cytokines and plays a core role in tissue morphogenesis and maintenance. As an important member involved in inflammation and immunity, the main role of versican is to recruit and activate white blood cells. Versican can be synthesised by many different types of cells, such as epithelial cells, endothelial cells, stromal cells, and white blood cells, and its synthesis is regulated by a series of pro-inflammatory cytokines and growth factors [16, 17]. Studies have shown that versican interacts indirectly or directly with inflammatory cells through hyaluronic acid, such as CD44, p-selectin glycoprotein ligand 1 (PSGL-1), and Toll-like receptors (TLRs). These interactions activate signalling pathways and promote inflammatory cytokines such as TNF α, IL-6, and NF κ. The synthesis and secretion of versican also affects inflammation by interacting with various growth factors and cytokines that regulate inflammation, thereby affecting its bioavailability and biological activity [12].

The extracellular matrix, which is composed mainly of versican, can bind to different subpopulations of white blood cells. An in vitro study has found that blocking the ectopic accumulation of the extracellular matrix in human lung fibroblasts can significantly reduce the adhesion of monocytes [18]. An in vitro study of rat arterial smooth muscle cells showed that overexpression of the Versican V3 subtype significantly reduced the infiltration of macrophages and inhibited the filling of lipids, which showed that Versican V3 subtype played an anti-inflammatory role in atherosclerosis [19]. In addition, studies have shown that versican inhibits the synthesis of IL-10 by blocking the binding of hyaluronic acid and T lymphocytes, thereby reducing the immune suppression ability of cells [20].

Presently, the mechanism of action of Versican in bronchiectasis and its relationship with neutrophils have not been reported. Both healthy individuals and patients with bronchiectasis have a significant chemotactic effect on neutrophils in peripheral blood, which proved that versican plays an important role in the occurrence and development of bronchiectasis by inducing the migration of neutrophils. Chronic inflammation is the most important pathogenesis of tracheal dilation, and neutrophils are one of the most important inflammatory cells in chronic inflammation. Under the action of various neutrophil chemokines, neutrophils migrate and aggregate to the diseased tissue. In addition to phagocytosing pathogens, neutrophils also release various proteases such as neutrophil elastase (NE), matrix metalloproteinase (MMP), collagenase, and cathepsin G. If these proteases are continuously released in large quantities, they will disrupt the local protease inhibition system, causing excessive damage to lung tissue and migration of inflammation if the balance is disrupted. Proteases can participate in the ‘malignant vortex’ of bronchiectasis by promoting mucus secretion, degrading extracellular matrix, and influencing cell apoptosis [21]. Neutrophils can also release decompressed DNA strands by assembling histones and various proteolytic enzymes to form complexes and NETs. In addition to directly trapping and killing pathogens, excessive NETs can directly damage lung tissue and the airway matrix, activate acquired immunity, and further damage bronchi [22].

This study has limitations. First, current research results still require more clinical samples for validation. Second, there are few reports on the function and clinical significance of VCAN, and further experimental verification is needed for the specific molecular function. In future studies, we will continue to conduct in-depth research, integrate multi-centre and multi-omics data, and use methods such as animal models to deeply explore the pathogenesis of bronchiectasis.

In summary, our study confirms that the expression levels of VCAN mRNA and protein in patients with bronchiectasis are elevated, which may be involved in the occurrence and development of bronchiectasis by increasing the migration and infiltration of neutrophils. Therefore, VCAN may be a potential therapeutic target for bronchiectasis.

### Supplementary Information

**Supplementary Material 1.**

## Data Availability

The datasets used and/or analysed during the current study are available from the corresponding author on reasonable request.

## Abbreviations

CF: Cystic fibrosis
OPAT: Outpatient parenteral antibiotic therapy
VCAN: Versican
NET: Neutrophil extracellular trap
DMEM: Dulbecco’s Modified Eagle Medium
RT: qPCR Real-time quantitative polymerase chain reaction
ELISA: Enzyme-linked immunosorbent assay
*P.a*: *Pseudomonas aeruginosa*
MALDI-TOF: Matrix-assisted laser desorption/ionisation time-of-flight
mRNA: Messenger RNA
SMD: Standardised mean difference
ROC: Receiver operating characteristic
FBS: Foetal bovine serum
SD: Standard deviation
IQR: Interquartile range
GEO: Gene Expression Omnibus
MOI: Multiplicity of infection
PSGL-1: P-selectin glycoprotein ligand 1
TLR: Toll-like receptor
NE: Neutrophil elastase
MMP: Matrix metalloproteinase
